# Genetically predicted cognitive performance and risk of meniscal derangement: A 2-sample Mendelian randomization study

**DOI:** 10.1097/MD.0000000000049983

**Published:** 2026-07-31

**Authors:** Chao Li, Mengjie Zhang, Jie Yin, Diping Cao, Bingli Liu, Baoqing Yu

**Affiliations:** a Department of Joint Surgery, Seventh People’s Hospital of Shanghai University of Traditional Chinese Medicine, Shanghai, China; b Department of Respiratory Medicine, Putuo Hospital, Shanghai University of Traditional Chinese Medicine, Shanghai, China.

**Keywords:** cognitive performance, Mendelian randomization, meniscal derangement

## Abstract

The causal relationship between cognitive performance and meniscal derangement is unclear. This study aims to investigate the potential genetic association between cognitive performance and meniscal derangement. We conducted a 2-sample Mendelian randomization (MR) analysis utilizing summary-level data from extensive genome-wide association studies. Single nucleotide polymorphisms (SNPs) achieving genome-wide significance (*P* < 5 × 10^−8^) were employed as instrumental variables for each exposure. The inverse-variance weighted (IVW) method served as the principal statistical technique, complemented by the weighted median, MR-Egger regression, and MR-PRESSO methods for sensitivity analyses, accommodating some of the assumptions inherent in IVs. Genetically predicted cognitive performance was inversely correlated with the odds of meniscal derangement. However, the MR-Egger regression analysis did not show a statistically significant association. The IVW method yielded an odds ratio (OR) of 0.76 (95% CI: 0.66–0.88, *P* = .0002) per standard deviation increase in genetically predicted cognitive performance. The weighted median method showed a directionally consistent association (OR = 0.81, 95% CI: 0.67–0.98, *P* = .030). Additionally, no evidence of directional horizontal pleiotropy was detected using the MR-Egger intercept (*P* > .05). Significant heterogeneity was observed across SNP-specific estimates (*P* < .05). This 2-sample MR study suggests that higher genetically predicted cognitive performance is associated with a lower risk of meniscal derangement. Further studies are warranted to validate this association and clarify the underlying neurocognitive and musculoskeletal mechanisms.

## 1. Introduction

The knee joint is the primary weight-bearing joint in humans. The meniscus consists of 2 pieces of fibrocartilage located on the medial and lateral sides of the knee joint, situated between the femur and the tibia. Meniscal derangements are classified into traumatic and degenerative types. Traumatic injuries typically occur in young, active individuals, mainly due to major sports-related incidents or occupations involving physical activity. Conversely, degenerative injuries are common in middle-aged and elderly individuals and are often associated with osteoarthritis.

Cognitive impairment is a common chronic disease associated with aging, characterized by memory loss, inattention, difficulty accessing new information, and problem-solving abilities. Recent studies have indicated that the decline in cognitive ability increases the risk of cardiovascular diseases,^[1]^ and patients with rheumatoid arthritis also exhibit significant cognitive decline.^[2]^ However, research on the relationship between cognitive performance and meniscal derangement is lacking, and the potential relationship between them remains uncertain, necessitating further investigation.

Mendelian randomization (MR) provides a framework to integrate genetic and phenotypic data, using genetic variants as natural instruments to infer causal relationships. This study applies MR to explore the association between genetically predicted cognitive performance and the risk of meniscal derangement using summary-level genome-wide association study (GWAS) data. MR examines whether observed correlations between exposure variables and outcomes are compatible with causal effects by utilizing genetic variation as an instrumental variable (IV).^[3]^ This method helps avoid confounding variables and reverse causation and minimizes bias since genetic variation is unaffected by external environmental, social, behavioral, or other factors.^[4]^ Therefore, the aim of this study was to determine the potential causal link between cognitive performance and meniscal derangement using a 2-sample MR analysis.

## 2. Materials and methods

### 2.1. Study overview

This MR study utilized published single nucleotide polymorphisms (SNPs) from the GWAS database as instrumental variables to assess the impact of cognitive performance on the risk of meniscal derangement. The inverse variance weighted (IVW) method was primarily employed to infer the causal relationship between these exposures and meniscal derangement. For the MR analysis to be valid, 3 fundamental assumptions must be met: First, the selected SNPs should be significantly associated with cognitive performance. Second, these SNPs must not be linked to any potential confounders that could affect the association between cognitive performance and meniscal derangement. Finally, the SNPs should not be directly associated with meniscal derangement itself, ensuring that any causal inference is mediated exclusively through cognitive performance. Institutional review board approval was not required for this work as the data used in this research are publicly available and were obtained with corresponding ethical approval.

### 2.2. Data source

Summary-level GWAS data for cognitive performance were obtained from the IEU OpenGWAS database. The exposure dataset was derived from the GWAS meta-analysis by Lee et al^[5]^ and is available under OpenGWAS ID ebi-a-GCST006572. This GWAS included 2,57,841 participants of European ancestry and defined cognitive performance as a general cognitive ability-related phenotype based on cognitive test performance in the contributing cohorts. Cognitive performance was treated as a continuous exposure trait in the present Mendelian randomization analysis. Summary-level GWAS data for meniscal derangement were obtained from the IEU OpenGWAS database by searching GWAS ID finn-b-M13_MENISCUSDERANGEMENTS. The outcome phenotype was meniscal derangement, rather than broad meniscal injury. This FinnGen-based GWAS included 13,568 cases and 1,47,221 controls of European ancestry, with 1,63,80,200 SNPs tested. The phenotype definition was based on registry-derived diagnostic codes for meniscal derangement, including ICD-10 codes M23.0–M23.3 and ICD-9 codes 7170–7175. Both exposure and outcome GWAS datasets were accessed on June 6, 2024. The exposure and outcome GWAS datasets were obtained from different GWAS sources. However, because only summary-level data were available, individual-level sample overlap could not be completely assessed. Therefore, potential sample overlap cannot be fully excluded and was acknowledged as a limitation.

### 2.3. Instrumental selection

From the pooled GWAS datasets, we first identified SNPs strongly associated with cognitive performance (*P* < 5 × 10^−8^). We then used a reference set of 1000 genomes from a European population to rule out linkage disequilibrium between these SNPs (*r*^2^ < 0.001 and clump window > 10,000 kb). Palindromic SNPs were manually eliminated.^[6]^ Following these procedures, the remaining SNPs were used as instrumental variables. To reduce weak instrument bias (*F* > 10), we also employed the *F*-statistic (*F* = β^2^/SE^2^) to assess the statistical efficacy of the SNPs and exclude those with poor statistical efficacy.^[7]^

### 2.4. Mendelian randomization analyses

All statistical analyses were conducted using R software (version 2024.04.1; R Foundation for Statistical Computing, Vienna, Austria) with the TwoSampleMR (0.6.3; MRC Integrative Epidemiology Unit, University of Bristol, Bristol, UK)^[8]^ and Mendelian randomization (version 0.6.3; MRC Biostatistics Unit, University of Cambridge, Cambridge, UK) packages,^[9]^ along with the MR-PRESSO package (available through the MR-PRESSO GitHub repository).^[10]^ *P*-values were 2-tailed throughout. Odds ratios (ORs) and the corresponding 95% confidence intervals (95% CIs) for meniscal derangement were scaled per 1-unit increase in genetically predicted exposure levels to cognitive performance. *F*-statistics for each exposure were calculated using the previously outlined approximation method.^[11]^

Our primary analytical strategy employed the standard IVW method under a random-effects model, assuming the validity of each SNP as an IV. While the IVW method synthesizes Wald ratios within a meta-analytic framework to yield precise association estimates, it remains susceptible to pleiotropic effects from invalid IVs.^[12]^ Variance among genetic variants was assessed using Cochran *Q*-statistic.^[12]^

To evaluate the assumed causal direction, we performed the MR Steiger directionality test. This test assessed whether the selected genetic instruments explained more variance in the exposure, cognitive performance, than in the outcome, meniscal derangement. A correct causal direction with a Steiger *P* value < .05 was considered supportive of the hypothesized direction from cognitive performance to meniscal derangement. To evaluate whether the selected genetic instruments were associated with potential confounders or horizontally pleiotropic traits, we performed a phenome-wide association search using the IEU OpenGWAS database through the ieugwasr package. We searched secondary phenotype associations of the instrumental SNPs at genome-wide significance. The original cognitive performance GWAS was excluded from the search results. We focused on prespecified potential confounders or risk-related traits, including educational attainment, socioeconomic status, body mass index, physical activity, osteoarthritis, knee-related disorders, musculoskeletal traits, diabetes, smoking, and alcohol consumption. SNPs associated with these prespecified traits were identified as potentially pleiotropic variants. A sensitivity analysis was then performed after excluding these SNPs to evaluate whether the main MR estimate was robust to potential confounder-related pleiotropy.

For sensitivity analysis, we used methods that relax certain IV assumptions, including the weighted median, MR-Egger regression, and MR-PRESSO. The weighted median method provides consistent estimates, assuming that the majority of the analysis weight comes from valid instruments.^[11]^ MR-Egger regression helps identify and adjust for directional pleiotropy, offering a pleiotropy-corrected causal estimate, though at a loss of statistical power.^[11]^ The *P*-value for the MR-Egger intercept was used to indicate the presence of pleiotropy.^[11]^ The MR-PRESSO method identifies outliers through a global test and recalculates estimates after outlier removal.^[10]^ The *P*-value from the MR-PRESSO distortion test indicated significant deviations in estimates before and after outlier adjustment.^[10]^

## 3. Results

### 3.1. Instrumental variables

After clumping and harmonization, 136 SNPs strongly associated with cognitive performance were selected as instrumental variables. The *F*-statistics of all SNPs were >10 (range: 29.79–116.11), indicating that the selected instrumental variables were very strong as a whole, and there was no weak instrumental variable problem.

### 3.2. Causal relationship between cognitive performance and meniscal derangement

The MR results of cognitive performance on meniscal derangement are presented in Table 1. Genetically predicted cognitive performance was inversely associated with the odds of meniscal derangement. The IVW method produced a pooled odds ratio (OR) of 0.76 (95% CI: 0.66–0.88, *P* = .0002) per standard deviation increase in cognitive performance, with a directionally consistent estimate from the weighted median method (OR = 0.81, 95% CI: 0.67–0.98, *P* = .030). Additionally, no pleiotropic effects were detected in our investigation using the MR-Egger intercept test (*P* > .05).

**Table 1:** Mendelian randomization (MR) estimates for the association between genetically predicted cognitive performance and the risk of meniscal derangement.

Exposure	MR method	OR (95% CI)	*P*
cognitive performance: 136 SNPs	MR Egger	0.95 (0.47, 1.91)	.89
	Weighted median	0.81 (0.67, 0.98)	.030
	Inverse variance weighted	0.76 (0.66, 0.88)	.0002
	Simple mode	0.86 (0.48, 1.52)	.61
	Weighted mode	0.88 (0.53, 1.48)	.65

CI = confidence interval, MR = Mendelian randomization, OR = odds ratio, SNP = single nucleotide polymorphism.

### 3.3. Sensitivity analysis

**Figure 1.**
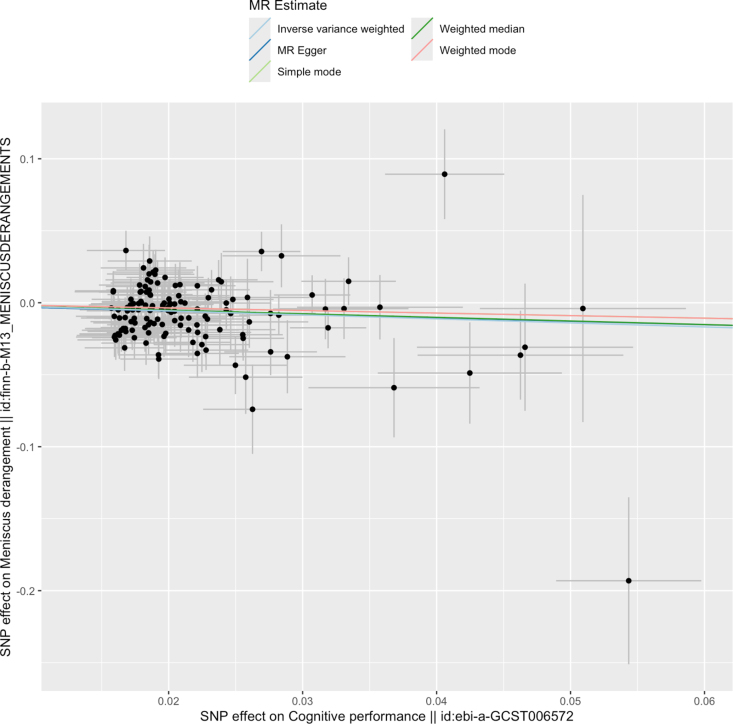
Scatter plots showing the effects of cognitive performance on the risk of meniscal derangement. Each point represents the per allele association with exposure plotted against per allele association with meniscal derangement. MR = Mendelian randomization, SNP = single nucleotide polymorphism.

**Figure 2.**
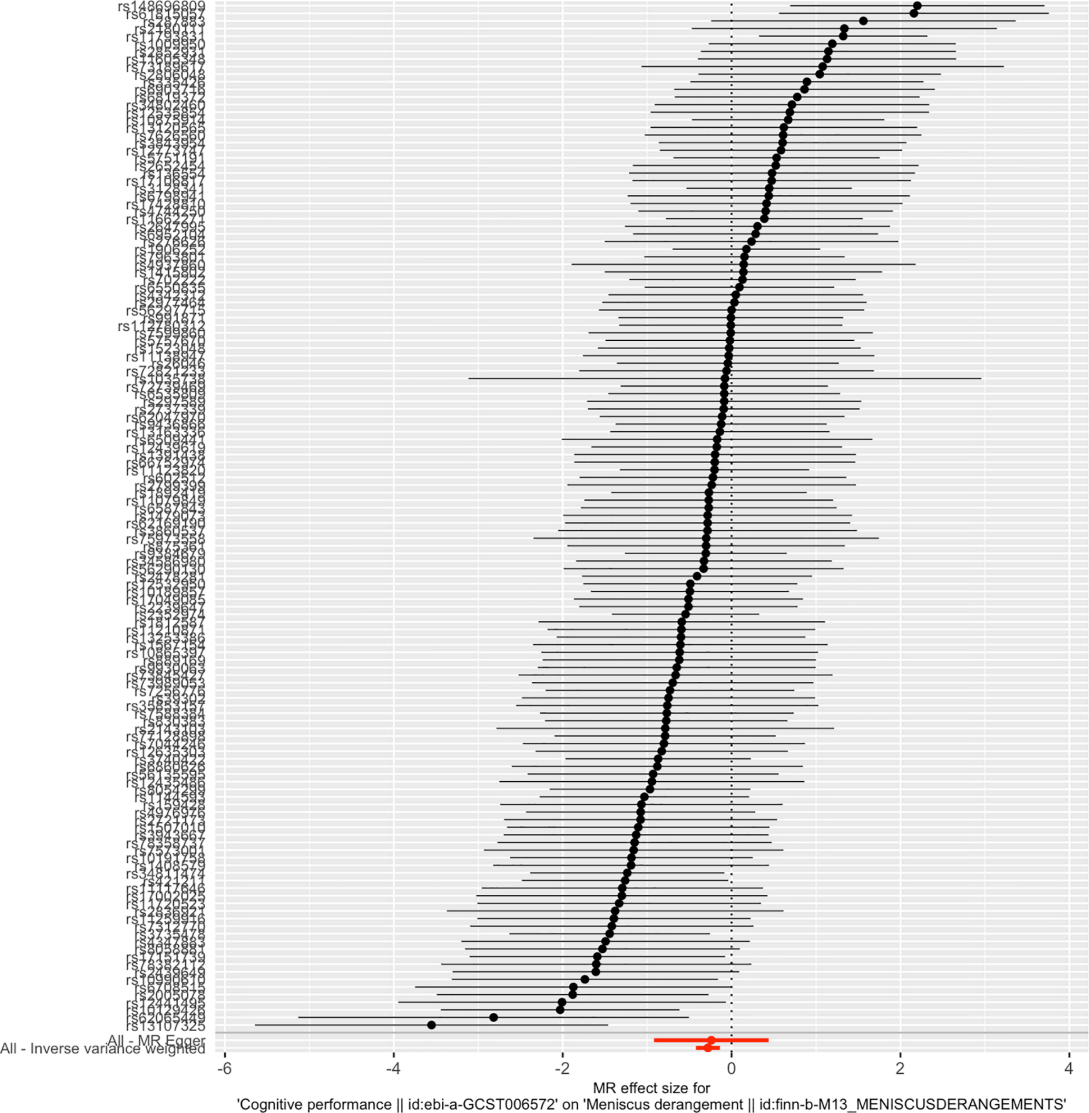
Forest plot showing Mendelian randomization effect estimates for the association between genetically predicted cognitive performance and the risk of meniscal derangement. MR = Mendelian randomization.

**Figure 3.**
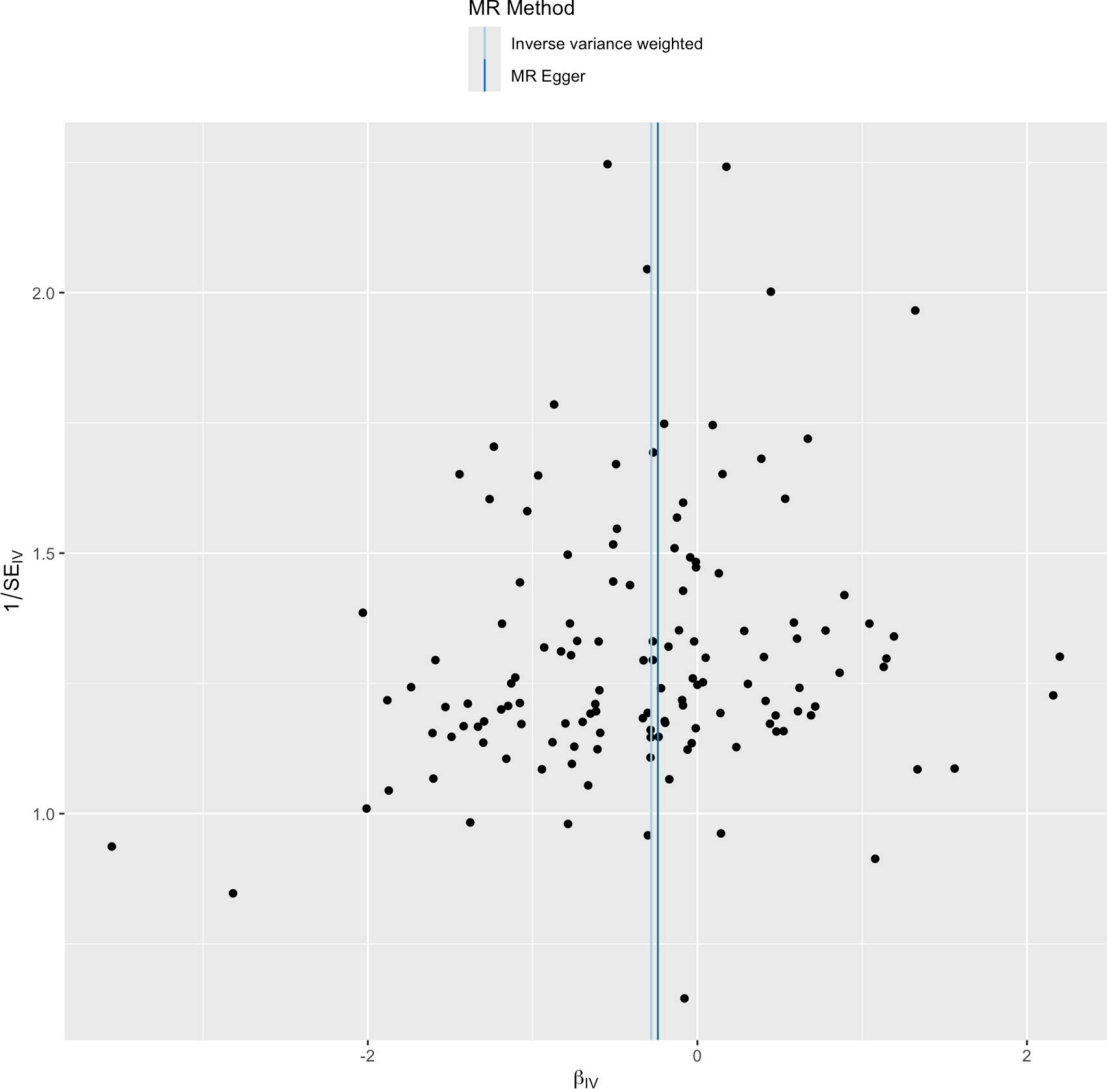
Funnel plot assessing the symmetry of SNP-specific causal estimates for genetically predicted cognitive performance and meniscal derangement. MR = Mendelian randomization, SE = standard error, SNP = single nucleotide polymorphism.

**Figure 4.**
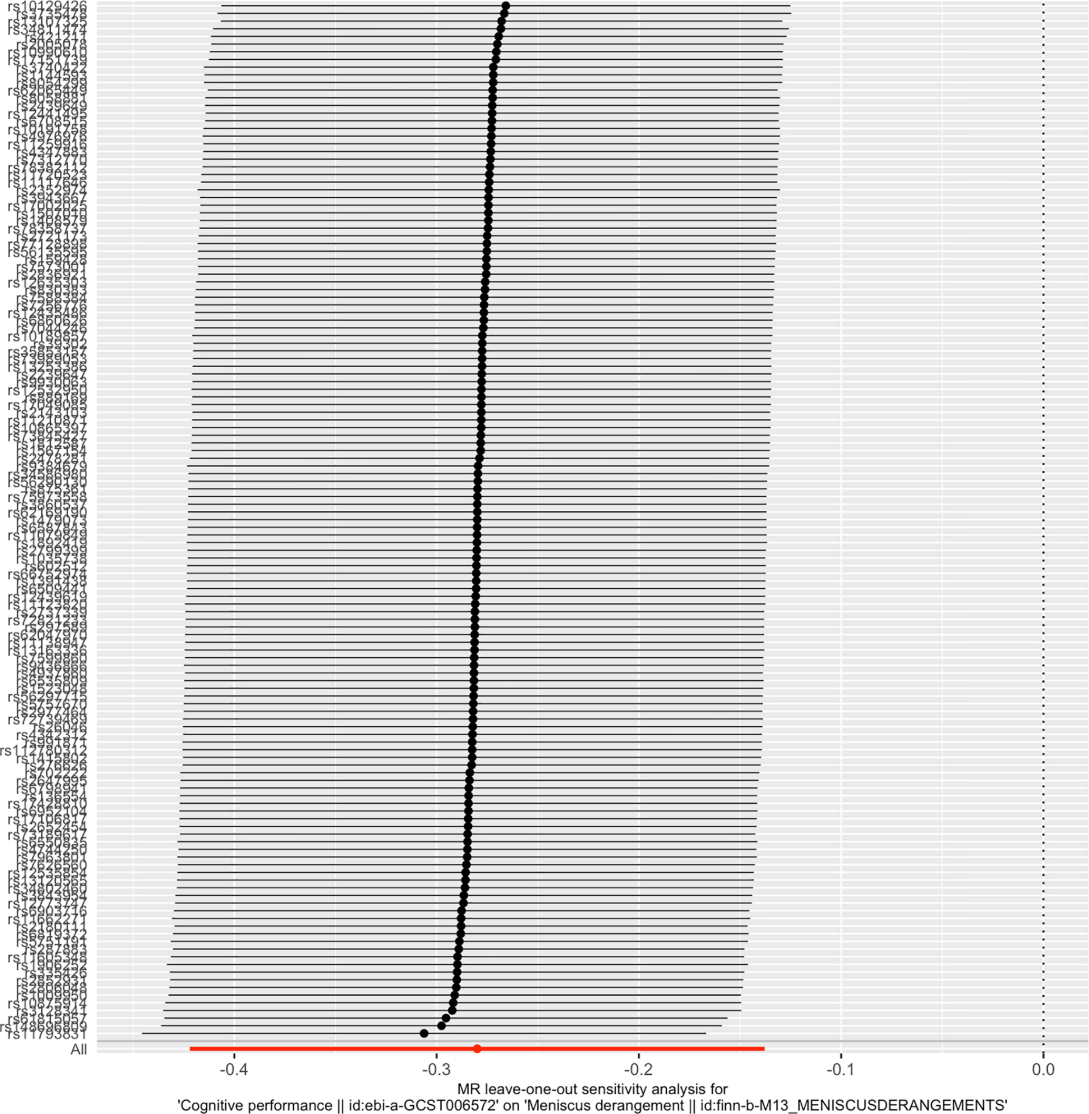
Leave-one-out analysis assessing whether the association between genetically predicted cognitive performance and meniscal derangement was driven by any single SNP. MR = Mendelian randomization, SNP = single nucleotide polymorphism.

The MR Steiger directionality test supported the hypothesized causal direction from cognitive performance to meniscal derangement. The selected genetic instruments explained more variance in cognitive performance than in meniscal derangement, suggesting that the observed association was unlikely to be explained by reverse causation. The Steiger directionality test showed a statistically significant result (Steiger *P* < .001). The detailed results of the MR Steiger directionality test are provided in Table S1, Supplemental Digital Content 1. There was heterogeneity among the results (Cochran *Q P* < .05). The above sensitivity analysis results are shown in Table 2. Figure 1 illustrates the scatter plots showing the associations between genetically predicted cognitive performance and the risk of meniscal derangement. The causal associations depicted in the forest plots are presented in Figure 2. To further validate these findings, the symmetry of the causal effect distribution was assessed through a funnel plot, which indicated no apparent bias from potential confounders, as shown in Figure 3. Based on the results of the heterogeneity test, we conducted MR-PRESSO analysis to detect and exclude outlier SNPs. The parameter NbDistribution was set to 10,000, and the SignifThreshold parameter was adjusted to 0.1. No outlier SNPs were identified in this process. Additionally, Leave-One-Out Analysis was performed to assess the excessive influence of individual SNPs on the overall results, confirming that no single SNP dominated the findings (Fig. 4). These sensitivity analyses suggested that the main finding was not driven by a single SNP or obvious directional pleiotropy, although heterogeneity remained present.

**Table 2:** Sensitivity analyses for the Mendelian randomization association between genetically predicted cognitive performance and meniscal derangement.

Exposure	Outcomes	Heterogeneity test				Pleiotropy test	
		MR-Egger		IVW		MR-Egger intercept	
		*Q*	*P*val	*Q*	*P*val	Intercept	*P*
Cognitive performance	Meniscus derangement	171.32	.01	171.8	.01	−0.004	.54

IVW = inverse-variance weighted, MR = Mendelian randomization.

To further assess potential horizontal pleiotropy through confounder-related variants, we performed a phenome-wide association search of the selected instrumental SNPs using the IEU OpenGWAS database. After excluding the original cognitive performance GWAS, 5891 secondary SNP–phenotype association records were identified. Among them, 511 records were related to prespecified potential confounders, including educational attainment, socioeconomic status, body mass index, physical activity, osteoarthritis, knee-related disorders, musculoskeletal traits, diabetes, smoking, and alcohol consumption, corresponding to 69 unique SNPs. The OpenGWAS PheWAS results for confounder-related SNP–phenotype associations are provided in Table S2, Supplemental Digital Content 2.

After excluding these 69 potentially pleiotropic SNPs, 72 SNPs remained for sensitivity analysis. The IVW estimate remained statistically significant and directionally consistent with the primary analysis (OR = 0.76, 95% CI: 0.63–0.92, *P* = .0047). The weighted median estimate showed a similar direction but did not reach statistical significance (OR = 0.82, 95% CI: 0.63–1.06, *P* = .133). These findings suggest that the observed inverse association was not entirely driven by SNPs associated with the prespecified potential confounders, although residual horizontal pleiotropy cannot be completely excluded. The MR results after excluding these potentially pleiotropic SNPs are provided in Table S3, Supplemental Digital Content 3.

## 4. Discussion

Previous observational evidence suggests that lower cognitive performance may be related to musculoskeletal outcomes, but the relationship between cognitive performance and meniscal derangement remains unclear. Our MR study aimed to investigate the potential association between genetically predicted cognitive performance and meniscal derangement. The primary IVW analysis suggested that higher genetically predicted cognitive performance was associated with a lower risk of meniscal derangement (OR = 0.76, 95% CI: 0.66–0.88, *P* = .0002). The weighted median analysis showed a directionally consistent association (OR = 0.81, 95% CI: 0.67–0.98, *P* = .030). However, the MR-Egger estimate did not reach statistical significance. Significant heterogeneity was observed across SNP-specific estimates. This heterogeneity may reflect the polygenic architecture of cognitive performance, differences in biological pathways represented by the selected variants, heterogeneity in the registry-based definition of meniscal derangement, or residual associations with behavioral, metabolic, socioeconomic, or musculoskeletal traits. Although random-effects IVW, MR-PRESSO, leave-one-out analysis, and OpenGWAS PheWAS-based sensitivity analysis were performed, heterogeneity may still affect the precision of causal inference. Therefore, the findings should be interpreted cautiously. Furthermore, statistical evidence from sensitivity analyses provides directionally consistent evidence. These findings may encourage future studies to consider cognitive and neuromuscular factors in risk assessment for meniscal derangement, particularly among individuals with cognitive impairment. However, the present MR evidence alone does not support direct changes to clinical screening or intervention strategies. Further clinical and mechanistic studies are needed to determine whether cognitive function has practical value in the prevention, detection, or management of meniscal derangement.

The prevalence of meniscal derangement has been reported to be increasing.^[13]^ Both conservative and surgical approaches are commonly employed for treatment.^[14]^ In the case of minor meniscal derangement, conservative methods like rest, application of cold packs, pain relievers, and physical therapy may be effective.^[15]^ These techniques aid in alleviating pain and inflammation while promoting self-healing of meniscal derangement. Surgical intervention may become necessary for severe meniscal derangement characterized by extensive tears or limited joint functionality.^[16]^ Surgery aims to repair or remove damaged meniscal tissue in order to restore normal joint function. Various factors contribute to the occurrence of meniscal derangement, making it crucial to identify these risk factors in clinical practice.^[17]^ Recognizing the risk factors associated with meniscal derangement assists physicians in evaluating patient susceptibility and devising personalized treatment plans. Previous research has highlighted several risk factors linked to meniscal derangement, including age, male gender, type of physical activity undertaken, higher body mass index, as well as delayed repair of accompanying anterior cruciate ligament injuries.^[18–22]^ Cognitive impairment predominantly affects the population over 65 years of age, with Alzheimer disease and vascular disease being the leading causes in the West.^[23]^ With an estimated 135 million people worldwide expected to have dementia by 2050,^[24]^ cognitive impairment poses a significant challenge to global health and social care.^[25]^

This study explored the potential association between genetically predicted cognitive performance and meniscal derangement. The findings suggest that higher genetically predicted cognitive performance may be associated with a lower risk of meniscal derangement, although this association requires further validation. Possible reasons include: First, cognitive decline may reduce athletic capacity and activity levels, increasing the risk of accidental falls and athletic injuries leading to meniscal derangement.^[26]^ Second, cognitive impairment often accompanies a decline in balance and coordination, raising the risk of injury during daily activities, particularly to the meniscus.^[27]^ Third, cognitive impairment may cause delays in attention and reaction time,^[28]^ elevating the risk of injuries in sports and daily activities. Fourth, cognitive impairment is often associated with other health problems, such as cardiovascular disease or diabetes,^[29,30]^ which may increase the risk of meniscal derangement. Additionally, some cognitive impairment treatments may affect physical abilities and balance. Fifth, cognitive impairment may lead to unhealthy lifestyles and behaviors,^[31-33]^ such as physical inactivity and poor diet, indirectly increasing the risk of meniscal derangement. A better understanding of the relationship between cognitive performance and meniscal derangement may help guide future studies on risk assessment and prevention, but further clinical evidence is required.

The underlying mechanism linking cognitive performance and meniscal derangement is unclear. The relationship between higher cognitive performance and reduced risk of meniscal derangement may involve mediation through interconnected neurocognitive-motor pathways and behavioral mechanisms. Enhanced cognitive capacities, particularly executive function and processing speed, are essential for dynamic decision-making during physically demanding tasks.^[34]^ Individuals with superior cognitive abilities demonstrate accelerated reaction times, facilitating rapid neuromuscular adjustments to mitigate injurious knee movements that predispose individuals to meniscal derangement.^[26]^ For example, delayed neuromuscular responses to uneven terrain or unexpected perturbations may amplify torsional forces on the meniscus, particularly in high-risk contexts such as athletic or occupational settings.^[27]^ Furthermore, cognitive impairment correlates with diminished neuromuscular coordination and postural control,^[35]^ potentially disrupting precise joint positioning during weight-bearing activities and promoting maladaptive loading patterns on meniscal tissue.^[36]^ Behavioral mediators constitute additional contributing factors: cognitive deficits are associated with sedentary behaviors, suboptimal adherence to injury prevention protocols, and comorbid conditions including obesity and cardiovascular disease.^[37-39]^ These factors may collectively heighten susceptibility to meniscal pathology through pathways involving chronic low-grade inflammation or accelerated joint degeneration.^[40]^

This study has several limitations. First, the analysis was based on summary-level GWAS data, which limited our ability to perform subgroup analyses by age, sex, injury subtype, disease severity, or treatment history. Second, both the exposure and outcome GWAS datasets were restricted to individuals of European ancestry, and the generalizability of the findings to other populations remains uncertain. Third, the outcome phenotype was meniscal derangement based on registry-derived GWAS data, which may not fully capture all clinical subtypes of meniscal pathology. Fourth, although multiple sensitivity analyses were performed, including MR-Egger, MR-PRESSO, leave-one-out analysis, MR Steiger directionality testing, and OpenGWAS PheWAS-based exclusion of potentially pleiotropic SNPs, residual horizontal pleiotropy and shared genetic architecture cannot be fully ruled out. Finally, MR analysis estimates the effect of genetically predicted lifelong differences in cognitive performance and should not be directly interpreted as evidence that short-term changes in cognitive function will alter the risk of meniscal derangement. In addition, because only summary-level GWAS data were available, individual-level sample overlap between the exposure and outcome datasets could not be completely assessed.

## 5. Conclusions

In conclusion, this 2-sample MR study suggests that higher genetically predicted cognitive performance is associated with a lower risk of meniscal derangement. Further clinical and mechanistic studies are needed to validate this association and clarify the underlying mechanisms.

## Acknowledgments

Data from a publicly available GWAS were used in this study, and the authors would like to thank all those who contributed and participated in the data collection.

## Author contributions

**Conceptualization:** Chao Li.

**Data curation:** Jie Yin.

**Formal analysis:** Jie Yin.

**Funding acquisition:** Bingli Liu, Baoqing Yu.

**Investigation:** Mengjie Zhang.

**Methodology:** Mengjie Zhang.

**Project administration:** Mengjie Zhang, Diping Cao.

**Resources:** Diping Cao.

**Software:** Chao Li, Diping Cao.

**Supervision:** Diping Cao.

**Validation:** Chao Li, Diping Cao.

**Writing – original draft:** Chao Li.

**Writing – review & editing:** Bingli Liu, Baoqing Yu.







## References

[1] Duan L Xiao R Liu S Shi Y Feng Y. Causality between cognitive performance and cardiovascular disease: a bidirectional Mendelian randomization study. Gene. 2024;891:147822. 37758004 10.1016/j.gene.2023.147822

[2] Duan L Li S Li H Shi Y Xie X Feng Y. Causality between rheumatoid arthritis and the risk of cognitive impairment: a Mendelian randomization study. Arthritis Res Ther. 2024;26:5. 38167504 10.1186/s13075-023-03245-x PMC10759661

[3] Emdin CA Khera AV Kathiresan S. Mendelian randomization. JAMA. 2017;318:1925–6. 29164242 10.1001/jama.2017.17219

[4] Ebrahim S Davey Smith G. Mendelian randomization: can genetic epidemiology help redress the failures of observational epidemiology? Hum Genet. 2008;123:15–33. 18038153 10.1007/s00439-007-0448-6

[5] Lee JJ Wedow R Okbay A.; 23andMe Research Team. Gene discovery and polygenic prediction from a genome-wide association study of educational attainment in 1.1 million individuals. Nat Genet. 2018;50:1112–21. 30038396 10.1038/s41588-018-0147-3 PMC6393768

[6] Hartwig FP Borges MC Horta BL Bowden J Davey Smith G. Inflammatory biomarkers and risk of schizophrenia: a 2-sample Mendelian randomization study. JAMA Psychiatry. 2017;74:1226–33. 29094161 10.1001/jamapsychiatry.2017.3191 PMC6583386

[7] Pierce BL Ahsan H Vanderweele TJ. Power and instrument strength requirements for Mendelian randomization studies using multiple genetic variants. Int J Epidemiol. 2011;40:740–52. 20813862 10.1093/ije/dyq151 PMC3147064

[8] Hemani G Zheng J Elsworth B. The MR-Base platform supports systematic causal inference across the human phenome. Elife. 2018;7:e34408. 29846171 10.7554/eLife.34408 PMC5976434

[9] Yavorska OO Burgess S. MendelianRandomization: an R package for performing Mendelian randomization analyses using summarized data. Int J Epidemiol. 2017;46:1734–9. 28398548 10.1093/ije/dyx034 PMC5510723

[10] Verbanck M Chen CY Neale B Do R. Detection of widespread horizontal pleiotropy in causal relationships inferred from Mendelian randomization between complex traits and diseases. Nat Genet. 2018;50:693–8. 29686387 10.1038/s41588-018-0099-7 PMC6083837

[11] Bowden J Del Greco M F Minelli C Davey Smith G Sheehan NA Thompson JR. Assessing the suitability of summary data for two-sample Mendelian randomization analyses using MR-Egger regression: the role of the I2 statistic. Int J Epidemiol. 2016;45:1961–74. 27616674 10.1093/ije/dyw220 PMC5446088

[12] Burgess S Bowden J Fall T Ingelsson E Thompson SG. Sensitivity analyses for robust causal inference from Mendelian randomization analyses with multiple genetic variants. Epidemiology. 2017;28:30–42. 27749700 10.1097/EDE.0000000000000559 PMC5133381

[13] Urhausen AP Berg B Øiestad BE. Measurement properties for muscle strength tests following anterior cruciate ligament and/or meniscus injury: what tests to use and where do we need to go? A systematic review with meta-analyses for the OPTIKNEE consensus. Br J Sports Med. 2022;56:1422–31. 36113973 10.1136/bjsports-2022-105498

[14] Wei Y Sun H Gui T. The critical role of Hedgehog-responsive mesenchymal progenitors in meniscus development and injury repair. Elife. 2021;10:e62917. 34085927 10.7554/eLife.62917 PMC8177886

[15] Han Y. Application of tissue engineered nanomaterials in meniscus sports injury repair. Front Bioeng Biotechnol. 2022;10:905869. 35774060 10.3389/fbioe.2022.905869 PMC9237472

[16] de Roy L Warnecke D Hacker SP. Meniscus injury and its surgical treatment does not increase initial whole knee joint friction. Front Bioeng Biotechnol. 2021;9:779946. 34957074 10.3389/fbioe.2021.779946 PMC8702854

[17] Brophy RH Gefen AM Matava MJ Wright RW Smith MV. Understanding of meniscus injury and expectations of meniscus surgery in patients presenting for orthopaedic care. Arthroscopy. 2015;31:2295–300.e5. 26163308 10.1016/j.arthro.2015.05.003

[18] Dumont GD Hogue GD Padalecki JR Okoro N Wilson PL. Meniscal and chondral injuries associated with pediatric anterior cruciate ligament tears: relationship of treatment time and patient-specific factors. Am J Sports Med. 2012;40:2128–33. 22729621 10.1177/0363546512449994

[19] Magnussen RA Pedroza AD Donaldson CT Flanigan DC Kaeding CC. Time from ACL injury to reconstruction and the prevalence of additional intra-articular pathology: is patient age an important factor? Knee Surg Sports Traumatol Arthrosc. 2013;21:2029–34. 23334624 10.1007/s00167-013-2380-8 PMC3652911

[20] Millett PJ Willis AA Warren RF. Associated injuries in pediatric and adolescent anterior cruciate ligament tears: does a delay in treatment increase the risk of meniscal tear? Arthroscopy. 2002;18:955–9. 12426537 10.1053/jars.2002.36114

[21] Vaquero J Vidal C Cubillo A. Intra-articular traumatic disorders of the knee in children and adolescents. Clin Orthop Relat Res. 2005;432:97–106. 10.1097/01.blo.0000156002.16750.8d 15738809

[22] Vavken P Tepolt FA Kocher MS. Concurrent meniscal and chondral injuries in pediatric and adolescent patients undergoing ACL reconstruction. J Pediatr Orthop. 2018;38:105–9. 27177235 10.1097/BPO.0000000000000777

[23] Iadecola C Duering M Hachinski V. Vascular cognitive impairment and dementia: JACC Scientific Expert Panel. J Am Coll Cardiol. 2019;73:3326–44. 31248555 10.1016/j.jacc.2019.04.034 PMC6719789

[24] Ponjoan A Garre-Olmo J Blanch J. Epidemiology of dementia: prevalence and incidence estimates using validated electronic health records from primary care. Clin Epidemiol. 2019;11:217–28. 30881138 10.2147/CLEP.S186590 PMC6407519

[25] Livingston G Sommerlad A Orgeta V. Dementia prevention, intervention, and care. Lancet. 2017;390:2673–734. 28735855 10.1016/S0140-6736(17)31363-6

[26] Riva D Fani M Benedetti MG Scarsini A Rocca F Mamo C. Effects of high-frequency proprioceptive training on single stance stability in older adults: implications for fall prevention. Biomed Res Int. 2019;2019:2382747. 31240206 10.1155/2019/2382747 PMC6556312

[27] Xiao T Yang L Smith L. Correlation between cognition and balance among middle-aged and older adults observed through a Tai Chi intervention program. Front Psychol. 2020;11:668. 32328017 10.3389/fpsyg.2020.00668 PMC7153433

[28] Sapsford TP Johnson SR Headrick JP. Forgetful, sad and old: do vascular cognitive impairment and depression share a common pre-disease network and how is it impacted by ageing? J Psychiatr Res. 2022;156:611–27. 36372004 10.1016/j.jpsychires.2022.10.071

[29] Sumbul-Sekerci B Hanagasi HA Bilgic B Tufekcioglu Z Gurvit H Emre M. Medication management and treatment adherence in Parkinson’s disease patients with mild cognitive impairment. Acta Neurol Belg. 2023;123:823–9. 35325434 10.1007/s13760-022-01916-1

[30] Khandaker GM Zuber V Rees JMB. Correction: Shared mechanisms between coronary heart disease and depression: findings from a large UK general population-based cohort. Mol Psychiatry. 2021;26:3659–61. 32807844 10.1038/s41380-020-0857-7 PMC8505250

[31] Coelho-Júnior HJ Trichopoulou A Panza F. Cross-sectional and longitudinal associations between adherence to Mediterranean diet with physical performance and cognitive function in older adults: a systematic review and meta-analysis. Ageing Res Rev. 2021;70:101395. 34153553 10.1016/j.arr.2021.101395

[32] Scarmeas N Luchsinger JA Schupf N. Physical activity, diet, and risk of Alzheimer disease. JAMA. 2009;302:627–37. 19671904 10.1001/jama.2009.1144 PMC2765045

[33] Damluji AA Ijaz N Chung SE. Hierarchical development of physical frailty and cognitive impairment and their association with incident cardiovascular disease. JACC Adv. 2023;2:100318. 37538136 10.1016/j.jacadv.2023.100318 PMC10399211

[34] Diamond A. Executive functions. Annu Rev Psychol. 2013;64:135–68. 23020641 10.1146/annurev-psych-113011-143750 PMC4084861

[35] Yogev-Seligmann G Hausdorff JM Giladi N. The role of executive function and attention in gait. Mov Disord. 2008;23:329–42; quiz 472. 18058946 10.1002/mds.21720 PMC2535903

[36] Roos EM Arden NK. Strategies for the prevention of knee osteoarthritis. Nat Rev Rheumatol. 2016;12:92–101. 26439406 10.1038/nrrheum.2015.135

[37] Sofi F Valecchi D Bacci D. Physical activity and risk of cognitive decline: a meta-analysis of prospective studies. J Intern Med. 2011;269:107–17. 20831630 10.1111/j.1365-2796.2010.02281.x

[38] Gopinath B Kifley A Flood VM Mitchell P. Physical activity as a determinant of successful aging over ten years. Sci Rep. 2018;8:10522. 30002462 10.1038/s41598-018-28526-3 PMC6043510

[39] Khandaker GM Zuber V Rees JMB. Shared mechanisms between coronary heart disease and depression: findings from a large UK general population-based cohort. Mol Psychiatry. 2020;25:1477–86. 30886334 10.1038/s41380-019-0395-3 PMC7303009

[40] Sellam J Berenbaum F. Is osteoarthritis a metabolic disease? Joint Bone Spine. 2013;80:568–73. 24176735 10.1016/j.jbspin.2013.09.007

